# Inhibition of neuroinflammation by GIBH-130 (AD-16) reduces neurodegeneration, motor deficits, and proinflammatory cytokines in a hemiparkinsonian model

**DOI:** 10.3389/fnana.2024.1511951

**Published:** 2024-12-16

**Authors:** Maria E. Bianchetti, Ana Flavia F. Ferreira, Luiz R. G. Britto

**Affiliations:** Department of Physiology and Biophysics, Institute of Biomedical Sciences, University of São Paulo, São Paulo, Brazil

**Keywords:** neurodegeneration, neuroprotection, neuroinflammation, microglia, Parkinson’s disease, 6-hydroxydopamine, cytokine profile

## Abstract

Parkinson’s disease (PD) is a neurodegenerative condition characterized by the loss of dopaminergic neurons in the substantia nigra pars compacta (SNc) of the brain, manifesting itself with both motor and non-motor symptoms. A critical element of this pathology is neuroinflammation, which triggers a harmful neurotoxic cycle, exacerbating cell death within the central nervous system. AD-16 (also known as GIBH-130) is a recently identified compound capable of reducing the expression of pro-inflammatory cytokines while increasing the expression of anti-inflammatory cytokines in Alzheimer’s disease models. Here, for the first time, we sought to comprehend the potential impact of orally administered AD-16 in mitigating neurodegeneration and subsequent disease progression in PD. To accomplish this, 6- hydroxydopamine (6-OHDA) unilateral striatal injections were employed to induce a PD model in male C57BL/6 mice. Cylinder and apomorphine-induced rotation behavior tests were conducted to assess motor behavior and validate the PD model 3 days after the injection. AD-16 was administered via gavage daily between days 3 and 9 after surgery. On the last day of treatment, motor tests were performed again. All animals were euthanized on day 10 and immunohistochemistry techniques were performed to detect tyrosine hydroxylase (TH) and Iba-1 and thus label dopaminergic neurons and microglia in the SNc and striatum (CPu). These same regions were collected for ELISA assays to assess different cytokine concentrations. Our results revealed an enhancement in the motor function of the AD-16-treated animals, as well as reduced nigrostriatal neurodegeneration. In addition, AD-16 reduced the increase in microglia density and prevented the changes in its morphology observed in the PD animal models. Furthermore, AD-16 was able to avoid the increase of pro-inflammatory cytokines levels that were present in 6-OHDA-injected animals who received vehicle. Consequently, AD-16 emerges as a compound with significant potential for negative modulation of neurodegeneration and neuroinflammation suppression in the 6-OHDA animal model of Parkinson’s disease.

## Graphical Abstract

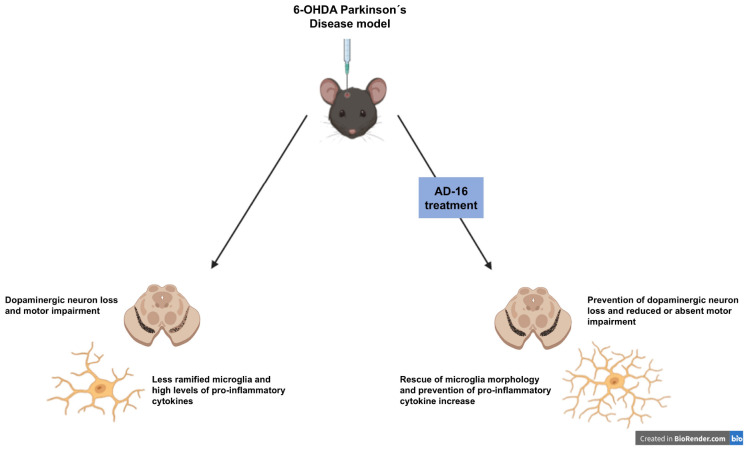

## 1 Introduction

Parkinson’s disease (PD) is the second most common neurodegenerative disease and the most common progressive motor disorder. Patients affected with PD typically present motor symptoms, being tremors, rigidity, bradykinesia, and postural instability the most typical ones (Poewe et al., 2017). Non-motor symptoms including constipation, urinary problems, sleep disorders, pain, and cognitive deficits are also reported in patients with this devastating disease (Schapira et al., 2017). Under the physiopathology of the disease, two main aspects can be noted: the degeneration of dopaminergic neurons from the substantia nigra pars compacta (SNc) and a consequent reduction of dopamine release and the presence of Lewy bodies, which are aggregates of alpha-synuclein (Dauer and Przedborski, 2003). Motor signs become evident when around 40–60% of the dopaminergic neurons have been lost (Riederer and Wuketich, 1976; Cheng et al., 2010) and the available treatments have several limitations (di Biase et al., 2023), which reinforce the urgent need for new therapies.

Recently, many have suggested neuroinflammation as a therapeutic target for PD (Lee et al., 2019). The neuroinflammatory response has physiological beneficial effects as it contributes, for instance, to tissue repair, axonal regeneration, and pathogen removal (Yong et al., 2019). However, an excessive inflammatory response leads to the release of neurotoxic factors, promotes tissue damage, and ultimately contributes to neurodegeneration (Zhang et al., 2023). Chronic neuroinflammation is a hallmark of several neurodegenerative diseases, including PD (Giri et al., 2024). In one of the first studies on this topic, reactive microglia were detected in the substantia nigra of PD patients (McGeer et al., 1988). After that, others have confirmed the critical role of neuroinflammation in PD, with increased inflammatory cytokine levels (Qin et al., 2016), oxidative stress (Wei et al., 2018), and other immune cells (Grozdanov et al., 2014) being reported in patients.

Microglia, the resident immune cell of the central nervous system, is one of the main players involved in neuroinflammation (Wang et al., 2015). Under physiological conditions, microglia participate in the defense against pathogens, the pruning of synapses, the promotion of neuronal survival and neuroplasticity, and the secretion of a variety of trophic factors, chemokines, and cytokines, among other functions (Isik et al., 2023). This cell type can assume heterogeneous morphological configurations, going from a homeostatic condition, characterized by a ramified morphology with many branches that help to monitor the surrounding environment, to an ameboid morphology with branch reduction, release of pro-inflammatory cytokines, and a more phagocytic profile (Vidal-Itriago et al., 2022). Under a pathological condition, as in PD, microglia become dysregulated, contributing to an inflammatory environment and enhancing neurodegeneration (Wang et al., 2015). Thus, modulating microglia might represent a potential therapeutical target for PD.

In 2016, in a microglia-based phenotypic screening, a new compound was found, named GIBH-130, later designated as AD-16 (Zhou et al., 2016). In this study, authors described the anti-neuroinflammatory effects of the compound by suppressing proinflammatory cytokine production, including IL-1β, TNF-α, and NO, in activated microglia. Using an animal model for Alzheimer’s disease, it was also reported that AD-16 improved short-term and long-term memory, restored the IL-4 and IL-6 levels in the brain, and protected neurons (Zhou et al., 2016). Others have also described the protective effects of AD-16 treatment by modulating neuroinflammation in Alzheimer’s disease (Sun et al., 2020), neonatal hypoxic-ischemic brain injury (Huang et al., 2022), and adult cerebral ischemia (Zhang et al., 2024) animal models. In addition, healthy subjects were treated with AD-16 in a randomized phase 1 study and the compound demonstrated safety, tolerability, and favorable pharmacokinetics (Peng et al., 2023). These studies indicate the potential of AD-16 as a therapeutic drug by modulating neuroinflammation. However, no one has evaluated before the effects of AD-16 in PD. In the present study, we aimed to investigate the AD-16 treatment effects on neurodegeneration and microglia activation in the 6-hydroxydopamine (6-OHDA) mouse model of PD.

## 2 Materials and methods

### 2.1 Animals

Experimental procedures were performed following the ARRIVE guidelines, the National Institutes of Health guide for the care and use of Laboratory animals (NIH Publications No. 8023, revised 1978), the National Council for the Control of Animal Experimentation (CONCEA, Brazil) guidelines, and the Ethics Committee for Animal Research of the Institute of Biomedical Sciences of the University of São Paulo (CEUA-ICB/USP, Brazil, protocol number: 6567270123). Three-month-old male C57BL/6 mice were provided by the facility for SPF mice production at USP Medical School. The mice were housed in an acclimatized facility on a 12-h light/dark schedule, with *ad libitum* access to standard chow and water.

### 2.2 Experimental design

**FIGURE 1:**
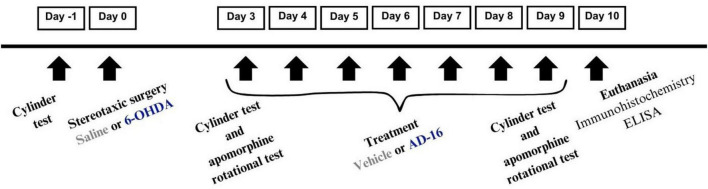
Experimental design demonstrating the schedule for 6-OHDA administration, AD-16 treatment, and behavior tests.

The animals were randomly divided into the following four groups (*n* = 12/group): (1) animals were given a saline injection into the right striatum (CPu) and vehicle orally (P.O.) (SAL + VEH); (2) animals were given a 6-OHDA injection into the right CPu and vehicle P.O. (6-OHDA + VEH); (3) animals were given a saline injection into the right CPu and AD-16 P.O. (SAL + AD-16); and (4) animals were given a 6-OHDA injection into the right CPu and AD-16 P.O. (6-OHDA + AD-16). Ten days after the PD model induction, animals were euthanized for brain tissue collection. Figure 1 illustrates the experimental approach.

### 2.3 Parkinson’s disease model induction

The PD model was induced by the administration of the neurotoxin 6-OHDA, a well-known animal model for this disease (Ferreira et al., 2024a). In brief, animals were anesthetized with isoflurane mixed with oxygen (5% for induction and 2% for maintenance, 0.8 L/min) and placed into a stereotaxic frame (David Kopf Instruments^®^). Stereotactic surgery was performed in which animals received a unilateral injection of 1 μL/10 μg of 6-OHDA (H4381, Sigma-Aldrich^®^) diluted in 0.3% ascorbic acid in saline or 1 μL of 0.3% ascorbic acid in saline alone into the right CPu. The coordinates were referenced from the Paxinos and Watson atlas: anteroposterior (AP) - 0.4 mm; lateral (L) - 2.0 mm; and ventral (V) - 3.0 mm, relative to the bregma. Injections were carried out using a Hamilton syringe (Neuros Syringes—65,460–02) at a rate of 0.5 μL/min, with the needle held at the injection site for an extra 5 min to prevent reflux. Analgesia was provided with meloxicam (2 mg/kg, s.c) and animals were observed until fully recovered (Ferreira et al., 2022, 2024b,2024c). Postoperative care included recording of the weight and s.c injections of meloxicam once daily for the next three days. Two animals did not survive the surgical procedure.

### 2.4 AD-16 treatment

The AD-16 (first named GIBH-130, IC50 3.4 nM) or vehicle treatment started 3 days after the PD model induction and lasted for 7 days. On day 3, the treatment was given around 5 h after the end of the behavior tests. The compound (GIBH-130; Abcam, ab287029) was prepared according to the manufacturer’s instructions. Briefly, the AD-16 was dissolved in 2% dimethyl sulfoxide (DMSO, D8418, Sigma-Aldrich^®^) in 0.9% saline solution. One mg/kg (volume of 3.33 μl/g) of AD-16 or the same volume of vehicle (2% dimethyl sulfoxide in 0.9% saline solution) was daily given to each mouse by oral gavage. The dose and route of administration were selected according to previous studies; (Huang et al., 2022; Zhang et al., 2024). All animals were treated by the experimenter within a consistent time window.

### 2.5 Behavior tests

#### 2.5.1 Cylinder test

The cylinder test was performed as described previously (Ferreira et al., 2022). This test is commonly used to assess the unilateral motor deficit induced by the 6-OHDA administration. Animals were tested at three time-points: one day before the PD model induction (day −1), three days after the PD model induction and before the start of AD-16 or vehicle treatments (day 3), and nine days after the PD model induction (day 9). Animals (*n* = 8–10/group) were placed in an acrylic cylinder (25 cm × 12 cm) with mirrors placed around it. The recording of the animal behavior was carried out for 5 min. The contacts of the right and left forelimbs with the cylinder wall were quantified and used to calculate the percentage of use of the ipsilateral/right paw {[number of ipsilateral limb contact (right forelimb)/number of total limb contact (right and left forelimbs)] × 100} in the three assessed times. To calculate the ipsilateral forelimb contacts, values from each animal on day 3 and day 9 were normalized to their respective baseline values.

#### 2.5.2 Apomorphine-induced rotational test

The apomorphine-induced rotational test was performed as reported before (Ferreira et al., 2024b). In this test, animals are given a subcutaneous injection of apomorphine, a dopamine agonist that can evoke asymmetrical turns toward the contralateral side of the lesion (left side in this case). Animals were tested at two time-points: three days after the PD model induction and before the start of AD-16 or vehicle treatments (day 3), and nine days after the PD model induction (day 9). Animals (*n* = 8–10/group) were given 0.5 mg/kg of apomorphine (A4393, Sigma-Aldrich^®^) in a final volume of 100 μL and recorded for 30 min. The number of rotations was counted and used in statistical analysis and graphs.

### 2.6 Immunohistochemistry

The immunohistochemistry assay was performed as described before (Ferreira et al., 2022). On the tenth day, animals (*n* = 4–5/group) were anesthetized and a transcardiac perfusion with 0.9% saline followed by 4% paraformaldehyde solution (PFA) was performed. Brains were collected and post-fixed in 4% PFA for 4 h. After that, they were stored in a 30% sucrose solution dissolved in 0.1 M phosphate buffer (PB, pH 7.4). A sliding freezing microtome (Leica SM2000 R Sliding microtome, RRID:SCR_018456) was used to slice the brains (30 μm thickness). Slides containing CPu and substantia nigra pars compacta (SNc) were incubated overnight with anti-tyrosine hydroxylase (TH; Millipore Cat# MAB318, RRID:AB_2201526, 1:1000) and anti-ionized calcium-binding adaptor molecule 1 (Iba1; Abcam Cat# ab5076, RRID:AB_2224402, 1:1000). The sections were then washed and incubated with the appropriate secondary antibodies: donkey anti-goat (Jackson ImmunoResearch Labs Cat# 705–065-003, RRID:AB_2340396, 1:200), goat anti-mouse (Jackson ImmunoResearch Labs Cat# 115–065-003, RRID:AB_2338557, 1:200), for 2 h followed by another 2 h incubation with avidin–biotin complex (VECTASTAIN Elite ABC-Peroxidase Kit, Vector Laboratories, Cat# PK-7100) and labeled with 0.05% 3–3’-diaminobenzidine (DAB) and hydrogen peroxide 0.01% in PB. Tissues were mounted on gelatin-coated slides and protected with glass coverslips.

### 2.7 Image acquisition, processing, and analysis

Immunoassayed brain slices of the SNc and the CPu were visualized and captured (Nikon Eclipse E1000 light-field optical microscope, Nikon DXM1200 camera). At least three slices per region per animal were captured. Images were analyzed with Fiji free software (Fiji, RRID:SCR_002285) as reported before (Ferreira et al., 2024c). For TH analysis, a 10× objective was used and the number of positive cells in the SNc was counted using the Cell Counter plugin. To cover the anteroposterior extent of the SNc, the analyzed sections were taken between the planes −2.80 and −3.64 mm relative to the bregma. For TH analysis in the CPu, we used integrated density. Three images from each slice (on the top, lateral, and central sides of the structure) were analyzed to get a picture of the whole region. The analyzed sections were taken between the planes +1.10 and −0.20 mm relative to the bregma. For both regions, the ratio between the experimental/lesioned hemisphere and the control/intact hemisphere was calculated per slice. The ratio from the slices was then averaged for each animal. For Iba-1 analysis, a 20× objective was used and the integrated density was assessed in at least three slices per animal for both SNc and CPu. For both regions, the ratio between the experimental/lesioned hemisphere and the control/intact hemisphere was calculated per slice. The ratio from the slices was then averaged for each animal. A morphological analysis was also performed in Iba-1- positive cells following the protocol reported before (Ferreira et al., 2024b). Only images from the experimental hemisphere captured with the 40× objective were used (3–5 slices were analyzed per region per animal). In brief, images were opened in Fiji free software (Fiji, RRID:SCR_002285) and a series of steps were performed: (1) 8-bit and grayscale conversion; (2) contrast and brightness adjustment; (3) binarized and black and white conversion; (4) and skeletonize with the AnalyzeSkeleton (2D/3D) plugin. Results and Branch information outputs were trimmed in Excel. The endpoints and branch length sum were divided by the total number of somas to obtain endpoints/total number of cells and branch length/total number of cells. Immunohistochemistry data were normalized to the saline vehicle group (SAL + VEH).

### 2.8 Enzyme-linked immunosorbent assay (ELISA)

Cytokine measurements of IFNγ, IL-1α, IL-1β, IL-6, IL-10, and TNFα were performed using the multiplex enzyme-linked immunosorbent assay (ELISA) kit according to the manufacturer instructions (Q-Plex Mouse Cytokine Panel 1 HS (6-Plex), Cat#: 111349MS, Quansys Biosciences). In brief, the midbrain and the CPu (*n* = 4/group) were collected on day 10 and homogenized in RIPA buffer (2 M NaCl; 1 M Tris, pH 7.4; 0.5 M EDTA; 10% Triton X-100 (v/v); 10% sodium deoxycholate (w/v); 10% SDS (w/v); 1 M dithiothreitol) and centrifuged at 12,000 rpm twice. The supernatant was collected, and protein levels were measured by the Bradford method. Samples (20 μL/well in duplicate) and a calibrator curve were added to the plate and incubated. Incubations with detection mix, streptavidin HRP, and the chemiluminescent substrate followed. The plate was imaged on G:BOX Chemi XRQ and analyzed on Q-View™ Software. The results were obtained from the calibrator curve in pg/mL. Total brain homogenate protein concentrations were obtained in mg/mL and used to normalize the data and therefore obtain the cytokines concentration referred to the total amount of proteins in the samples (pg/mg).

### 2.9 Statistical analyses

The software Statistical Package for the Social Sciences (SPSS), version 23 (IBM^®^ SPSS^®^ Statistic, Chicago, IL), was used to analyze the data. The Two-way ANOVA test followed by the Bonferroni *post-hoc* test was performed. Statistical significance was considered for values of *P* ≤ 0.05. Graphs were plotted in GraphPad Prism^®^ software 6 (GraphPad Prism, RRID:SCR_002798) and are presented as mean and standard errors of the mean (SEM). Data can be obtained from the corresponding author upon reasonable request.

## 3 Results

### 3.1 AD-16 treatment relieves motor impairment in the 6-OHDA mouse model

**FIGURE 2:**
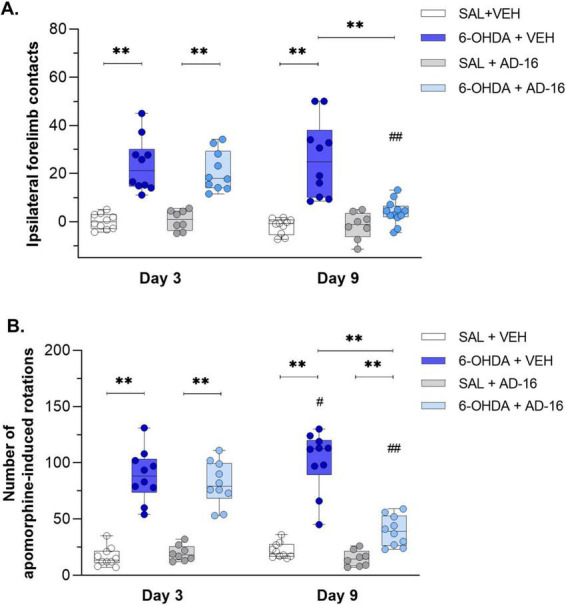
Behavioral test results. Difference of the right forelimb usage in relation to baseline assessed by the cylinder test **(A)** (SAL + AD-16: *n* = 8; 6-OHDA + VEH, SAL + VEH, and 6-OHDA + AD-16: *n* = 10). Number of asymmetric apomorphine-induced rotations assessed by the apomorphine **(B)** (SAL + AD-16: *n* = 8; 6-OHDA + VEH, SAL + VEH, and 6-OHDA + AD-16: *n* = 10). Data expressed as mean ± SEM. **p < 0.001; #*p* = 0.029; ##*p* < 0.001. *comparison between groups at the same time; #comparison within the same group across different times.

We first confirmed the PD model induction three days after 6-OHDA injections and before the beginning of the AD-16 treatment through motor behavioral tests (Figure 2). In the cylinder test [*F*(2, 68) = 8.524; *p* < 0.001], we observed an increase in the use of the ipsilateral forelimb (in this case the right paw) in animals that received the 6-OHDA-injection (SAL + VEH: 0.11 ± 1.01, 6-OHDA + VEH: 23.52 ± 3.53, *p* < 0.001; SAL + AD-16: 0.67 ± 1.48, 6-OHDA + AD-16: 20.91 ± 2.59, *p* < 0.001) (Figure 2A). Motor impairment was also confirmed in the apomorphine-induced rotational test [*F*(1, 34) = 14.320; *p* < 0.001]. Six-OHDA-injected animals presented an increase in the number of induced-rotations when compared to controls (SAL + VEH: 16 ± 3, 6-OHDA + VEH: 89 ± 7, *p* < 0.001; SAL + AD-16: 20 ± 2, 6-OHDA + AD-16: 82 ± 6, *p* < 0.001) (Figure 2B). These data indicate that the PD model was successfully induced.

After the model validation, AD-16 treatment started (day 3). By the end of the treatment (day 9) animals’ motor behavior was reassessed. In the cylinder test, while 6-OHDA-injected vehicle-treated animals showed increased ipsilateral forelimb contacts (SAL + VEH: −1.91 ± 1.05, 6-OHDA + VEH: 26.08 ± 5.01, *p* < 0.001) compared to control animals, the 6-OHDA-injected AD-16-treated group did not show difference from the control group (SAL + AD-16: −1.67 ± 1.98, 6-OHDA + AD-16: 3.98 ± 1.72, *p* = 0.201) (Figure 2A). In the apomorphine-induced rotational test, an increase in the 6-OHDA-injected vehicle-treated group was notice when compared to the control group (SAL + VEH: 22 ± 2, 6-OHDA + VEH: 102 ± 8, *p* < 0.001) and to the same group on day 3 (6-OHDA + VEH day 3: 89 ± 7, 6-OHDA + VEH day 9: 102 ± 8, *p* = 0.029) (Figure 2B), indicating the progression of the lesion as expected. On the other hand, 6-OHDA-injected AD-16-treated animals, despite still showing an increase in the number of induced rotations compared to the control animals (SAL + AD-16: 15 ± 2, 6-OHDA + AD-16: 39 ± 4, *p* = 0.003), presented a reduction in the number of rotations when compared to 6-OHDA-injected vehicle-treated animals (6-OHDA + VEH: 102 ± 8, 6-OHDA + AD-16: 39 ± 4, *p* < 0.001). These data indicate that the AD-16 treatment was able to alleviate the motor impairment induced by 6-OHDA.

### 3.2 Neurodegeneration is reduced after AD-16 treatment

**FIGURE 3:**
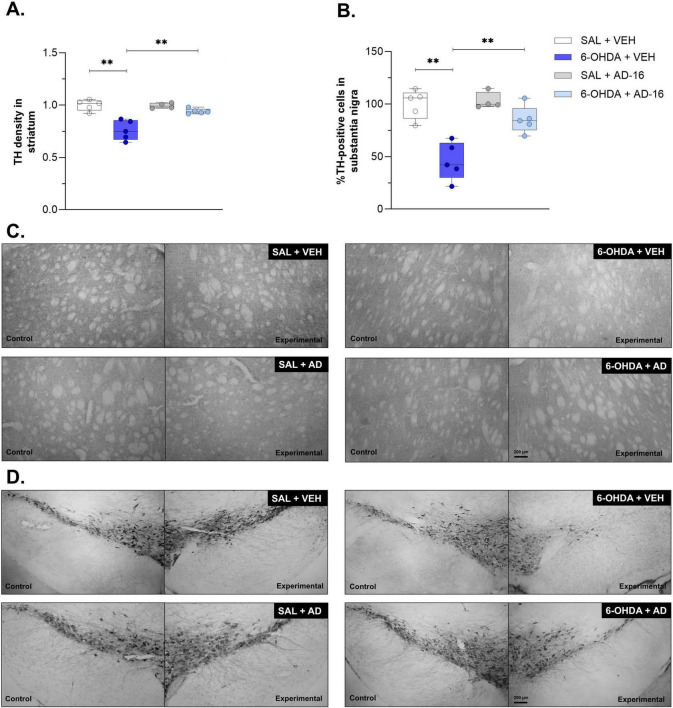
TH immunohistochemistry assay results. Comparison of TH density in the striatum and representative images **(A,C)** the percentage of TH-positive cells between animals in each group and representative images of the lesioned/experimental hemisphere and the intact/control hemisphere from the same animal **(B,D)** (SAL + AD-16: *n* = 4; 6-OHDA + VEH, SAL + VEH, and 6-OHDA + AD-16: *n* = 5). The images were not cropped. The scale bar represents 200 μm. Data expressed as mean ± SEM. **p < 0.001.

We next evaluated the neurodegeneration of dopaminergic neurons into SNc and dopaminergic terminals within CPu (Figure 3). The TH staining performed in the SNc revealed a decrease in the number of dopaminergic neurons in 6-OHDA-injected, vehicle-treated mice when compared to control mice [*F*(3, 14) = 6.490; *p* = 0.023] (SAL + VEH: 100 ± 6.65, 6-OHDA + VEH: 47.33 ± 8.30, *p* < 0.001) (Figure 3A). A reduction in TH density was also noticed in the CPu of 6-OHDA-injected animals [*F*(3, 14) = 10.365; *p* = 0.006] (SAL + VEH: 1.00 ± 0.03, 6-OHDA + VEH: 0.76 ± 0.04, *p* < 0.001) (Figure 3B). In 6-OHDA-injected, AD-16-treated animals the TH-positive cells number in the SNc and density in the CPu were not observed when compared to control animals (in the SNc, SAL + AD-16: 106.83 ± 4.12, 6-OHDA + AD-16: 88.60 ± 6.05, *p* = 0.077; in the CPu, SAL + AD-16: 1.00 ± 0.01, 6-OHDA + AD-16: 0.95 ± 0.01, *p* = 0.215) and increased when compared to 6-OHDA-injected, vehicle-treated animals (in the SNc, 6-OHDA + VEH: 47.33 ± 8.30, 6-OHDA + AD-16: 88.60 ± 6.05, *p* < 0.001; in the CPu, 6-OHDA + VEH: 0.76 ± 0.04, 6-OHDA + AD-16: 0.95 ± 0.01, *p* < 0.001). These findings indicate that AD-16 treatment was able to alleviate the PD model progression by preventing dopaminergic neuronal death.

### 3.3 Increase in microglia density and morphology alterations are alleviated after AD-16 treatment

**FIGURE 4:**
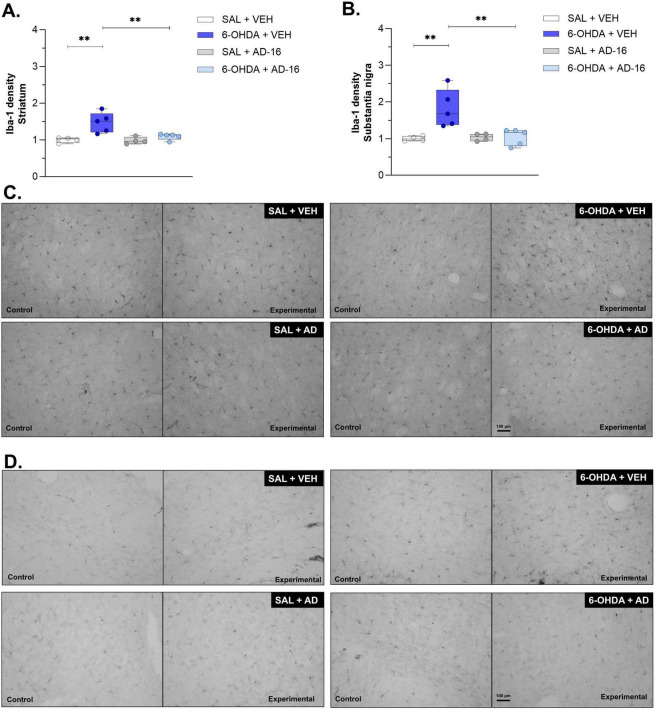
Iba-1 immunohistochemistry assay results. Comparison of Iba-1 density between animals in each group and representative images of the lesioned/experimental hemisphere and the intact/control hemisphere from the same animal in the striatum **(A,C)** and substantia nigra **(B,D)** (SAL + VEH and SAL + AD-16: *n* = 4; 6-OHDA + VEH and 6-OHDA + AD-16: *n* = 5). The images were not cropped. The scale bar represents 100 μm. Data expressed as mean ± SEM. **p = 0.001.

**FIGURE 5:**
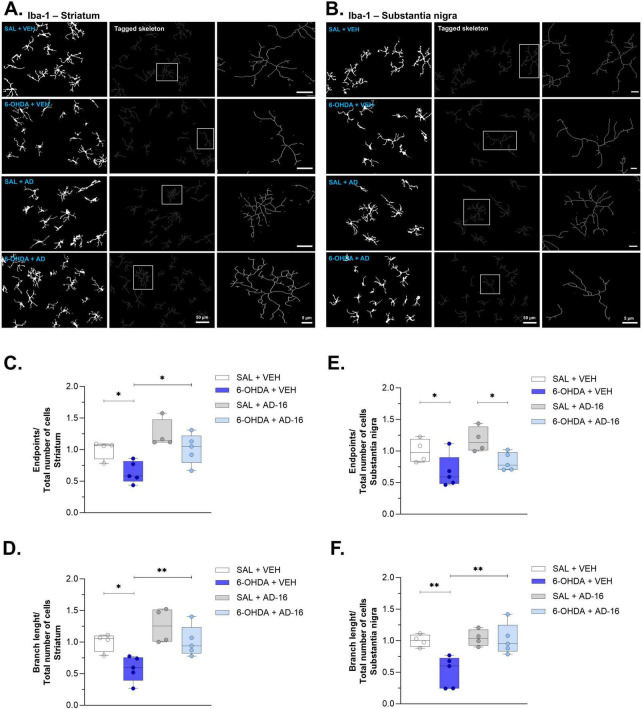
Iba -1 skeleton analysis results. Representative grayscale, tagged skeleton, and zoomed cell from the Skeleton analysis performed in the striatum **(A)**. Graphs of microglia endpoints/cell and branch length/cell, respectively, in the striatum (SAL + VEH and SAL + AD-16: *n* = 4; 6-OHDA + VEH and 6-OHDA + AD-16: *n* = 5) **(C,D)**. Representative grayscale, tagged skeleton, and zoomed cell from the Skeleton analysis performed in the substantia nigra **(B)**. Graphs of microglia endpoints/cell and branch length/cell, respectively, in the substantia nigra (SAL + VEH and SAL + AD-16: *n* = 4; 6-OHDA + VEH and 6-OHDA + AD-16: *n* = 5) **(E,F)**. Scale bars in the second column represent 50 μm. Scale bars in the third column (zoom image) represent 5 μm. Data expressed as mean ± SEM.*p < 0.04; **p < 0.005.

To further understand the role of AD-16 on microglia activation of the 6-OHDA mouse model, we first evaluated the density of Iba-1 in both the SNc and the CPu (Figure 4). The vehicle-treated 6-OHDA group showed an increase in Iba-1-staining in the CPu [*F*(3, 14) = 5.404; *p* = 0.036] (SAL + VEH: 1.00 ± 0.04, 6-OHDA + VEH: 1.47 ± 0.12, *p* = 0.001) (Figure 4A) and in the SNc [*F*(3, 14) = 7.866; *p* = 0.014] (SAL + VEH: 1.00 ± 0.03, 6-OHDA + VEH: 1.82 ± 0.23, *p* = 0.001) (Figure 4B) when compared to the control group. This increase was not observed in AD-16-treated animals, neither in the CPu (SAL + AD-16: 0.98 ± 0.05, 6-OHDA + AD-16: 1.09 ± 0.04, *p* = 0.324) nor in the SNc (SAL + AD-16: 1.04 ± 0.05, 6-OHDA + AD-16: 1.04 ± 0.10, *p* = 0.975). We next followed to investigate some morphological aspects of microglia by performing the Skeleton analysis (Figure 5). The number of endpoints and branch length were accessed in both regions. A reduction in the number of endpoints was observed in 6-OHDA-injected vehicle-treated mice in the CPu [*F*(3, 14) = 7.098; *p* = 0.004] (SAL + VEH: 1.00 ± 0.23, 6-OHDA + VEH: 0.64 ± 0.14, *p* = 0.018) (Figure 5C) and in the SNc [*F*(3, 14) = 5.101; *p* = 0.014] (SAL + VEH: 1.00 ± 0.24, 6-OHDA + VEH: 0.67 ± 0.16, *p* = 0.030) (Figure 5E). It was also noticed a decrease in branch length in this group on both the CPu [*F*(3, 14) = 7.390; *p* = 0.003] (SAL + VEH: 1.00 ± 0.23, 6-OHDA + VEH: 0.58 ± 0.13, *p* = 0.014) (Figure 5D) and the SNc [*F*(3, 14) = 7.985; *p* = 0.002] (SAL + VEH: 1.00 ± 0.23, 6-OHDA + VEH: 0.51 ± 0.14, *p* = 0.003) (Figure 5F). This indicates a reduction in microglia ramification. On the other hand, AD-16 treatment in 6-OHDA-injected animals presented a higher number of endpoints and longer branch when compared to vehicle-treated, 6-OHDA-injected animals in the CPu (Endpoints: 6-OHDA + VEH: 0.64 ± 0.14, 6-OHDA + AD-16: 1.01 ± 0.11; *p* = 0.011; Branch length: 6-OHDA + VEH: 0.58 ± 0.13, 6-OHDA + AD-16: 1.01 ± 0.11; *p* = 0.008) and a higher branch length in the SNc (Endpoints: 6-OHDA + VEH: 0.67 ± 0.16, 6-OHDA + AD-16: 0.83 ± 0.06; *p* = 0.237; Branch length: 6-OHDA + VEH: 0.51 ± 0.14, 6-OHDA + AD-16: 1.02 ± 0.11; *p* = 0.001).

### 3.4 Pro-inflammatory cytokine levels are attenuated after AD-16 treatment

**FIGURE 6:**
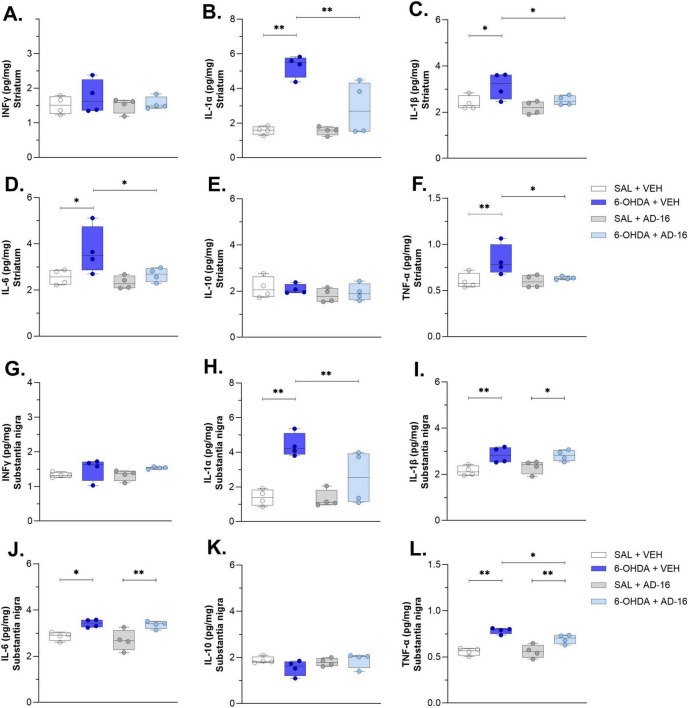
Cytokine ELISA assay results. IFNγ concentrations in the striatum **(A)** and the substantia nigra **(G)**. IL-1α concentrations in the striatum **(B)** and the substantia nigra **(H)**. IL-1β concentrations in the striatum **(C)** and the substantia nigra **(I)**. IL-6 concentrations in the striatum **(D)** and the substantia nigra **(J)**. IL-10 concentrations in the striatum **(E)** and the substantia nigra **(K)**. TNF-α concentrations in the striatum **(F)** and the substantia nigra **(L)**. *n* = 4/group. Data expressed as mean ± SEM. *p < 0.04; **p < 0.005.

Lastly, we accessed the cytokine levels of IFNγ, IL-1α, IL-1β, IL-6, IL-10, and TNF-α in the SNc and CPu (Figure 6). The production and secretion of pro-inflammatory, such as IFNγ, IL-1α, IL-1β, IL-6, and TNF-α, and anti-inflammatory, such as IL-10, cytokines have been studied and reported before in PD (Harms et al., 2021; Dzamko, 2023). Here we observed an increase in IL-1α, IL-1β, IL-6, and TNFα levels in both the SNc [IL-1α: *F*(3, 12) = 10.171; *p* = 0.001; IL-1β: *F*(3, 12) = 6.429; *p* = 0.008; IL-6: *F*(3, 12) = 7.196; *p* = 0.005; TNF-α: *F*(3, 12) = 18.814; *p* < 0.001] and the CPu [IL-1α: *F*(3, 12) = 17.251; *p* = 0.001; IL-1β: *F*(3, 12) = 4.993; *p* = 0.018; IL-6: *F*(3, 12) = 4.514; *p* = 0.024; TNF-α: *F*(3, 12) = 4.719; *p* = 0.021] when animals injected with 6-OHDA and treated with vehicle were compared to animals injected with saline and treated with vehicle (SNc: IL-1α: SAL + VEH: 1.39 ± 0.23, 6-OHDA + VEH: 4.40 ± 0.34, *p* < 0.001; IL-1β: SAL + VEH: 2.15 ± 0.11, 6-OHDA + VEH: 2.84 ± 0.17, *p* = 0.004; IL-6: SAL + VEH: 2.86 ± 0.09, 6-OHDA + VEH: 3.42 ± 0.08, *p* = 0.013; TNF-α: SAL + VEH: 0.56 ± 0.02, 6-OHDA + VEH: 0.78 ± 0.02, *p* < 0.001; CPu: IL-1α: SAL + VEH: 1.58 ± 0.12, 6-OHDA + VEH: 5.30 ± 0.32, *p* < 0.001; IL-1β: SAL + VEH: 2.40 ± 0.15, 6-OHDA + VEH: 3.14 ± 0.28, *p* = 0.014; IL-6: SAL + VEH: 2.55 ± 0.15, 6-OHDA + VEH: 3.70 ± 0.51, *p* = 0.015; TNF-α: SAL + VEH: 0.60 ± 0.04, 6-OHDA + VEH: 0.82 ± 0.08, *p* = 0.008). No difference was observed for IFNγ [SNc: *F*(3, 12) = 1.509; *p* = 0.262; CPu: *F*(3, 12) = 0.523; *p* = 0.675] and IL-10 [SNc: *F*(3, 12) = 1.489; *p* = 0.267; CPu: *F*(3, 12) = 0.741; *p* = 0.548]. These data confirmed the brain inflammatory environment induced by the 6-OHDA. We then assessed whether AD-16 could regulate this increase of inflammation. It was noted that AD-16 treatment reduced cytokines levels in the 6-OHDA-injected AD-16-treated group. In the CPu, IL-1α (6-OHDA + VEH: 5.30 ± 0.32; 6-OHDA + AD-16: 2.85 ± 0.77; *p* = 0.001), IL-1β (6-OHDA + VEH: 3.14 ± 0.28; 6-OHDA + AD-16: 2.51 ± 0.10; *p* = 0.031), IL-6 (6-OHDA + VEH: 3.70 ± 0.51; 6-OHDA + AD-16: 2.66 ± 0.15; *p* = 0.025), and TNF-α (6-OHDA + VEH: 0.82 ± 0.08; 6-OHDA + AD-16: 0.63 ± 0.01; *p* = 0.019) of 6-OHDA-injected AD-16-treated animals were found reduced compared to 6-OHDA-injected vehicle treated animals. In the SNc, despite the levels of IL-1β and IL-6 were still higher in the 6-OHDA-injected AD-16-treated group (IL-1β: 6-OHDA + VEH: 2.84 ± 0.17; 6-OHDA + AD-16: 2.81 ± 0.12; *p* = 0.896; IL-6: 6-OHDA + VEH: 3.42 ± 0.08; 6-OHDA + AD-16: 3.37 ± 0.08; *p* = 0.782), a decrease in IL-1α (6-OHDA + VEH: 4.40 ± 0.34; 6-OHDA + AD-16: 2.54 ± 0.76; *p* = 0.013) and TNF-α (6-OHDA + VEH: 0.78 ± 0.02; 6-OHDA + AD-16: 0.69 ± 0.03; *p* = 0.026) was observed in AD-16-treated animals. Overall, our results indicate that AD-16 treatment reduced pro-inflammatory cytokine levels in the brains of 6-OHDA-induced mice.

## 4 Discussion

Here we evaluated the effects of the anti-inflammatory compound, AD-16, on the 6-OHDA mouse model of PD. To do that, we chose a post-treatment approach that is initiated after the motor symptom confirmation, resembling what is observed in the patients. We found that AD-16 treatment was able to: (1) alleviate motor impairments; (2) reduce dopaminergic neuron death; and (3) attenuate inflammation by reducing microglia density, rescuing its morphology, and preventing the pro-inflammatory cytokine increase. These findings indicate that the modulation of neuroinflammation is indeed a potential tool for PD control and that AD-16 could represent a potential therapeutic approach for this disease.

The AD-16 compound was discovered among a series of molecules in 2016 in a screening that identified compounds able to suppress microglia activation (Zhou et al., 2016). It was shown that AD-16 reduced NO, TNF-α, and, more selectively, IL-1β production in lipopolysaccharide (LPS)-activated microglia. Besides that, the compound can cross the blood-brain barrier, does not present toxic effects, and has around 75% bioavailability and 4.32 h half-life when orally administered (Zhou et al., 2016). Recently, the pharmacokinetics, safety, and tolerability of AD-16 were observed in healthy subjects in a randomized, placebo-controlled study (Peng et al., 2023). The compound characteristics support its potential use as a drug treatment for inflammatory-related diseases, such as PD.

Others have evaluated the effects of AD-16 on Alzheimer’s disease and ischemia animal models. The compound was able to prevent the impairment in memory performances on two different models of Alzheimer’s disease (Zhou et al., 2016), reduce Aβ aggregates, a hallmark of Alzheimer’s disease (Sun et al., 2020), alleviate infarct volume and improve neurobehavioral outcomes in a neonatal hypoxic-ischemic model (Huang et al., 2022), and decrease brain infarct volume, brain edema, and neurological deficits in the adult cerebral ischemic injury mouse model (Zhang et al., 2024). Here, for the first time, we assessed the effects of AD-16 treatment in a PD animal model.

After intrastriatal injection with 6-OHDA to induce this model, there was a 53% decrease of TH-positive cells in the SNc and a 24% decrease of the TH staining density in the striatum of the PD group. It has been described that this model can cause moderate loss of TH density at striatal levels, followed by an increase in the lesion volume and subsequent loss of TH-positive cells in the SNc after several days (Farmer et al., 2020). While some studies have reported a larger loss in the striatum when compared to the SNc (Issy et al., 2015; Requejo et al., 2020), it is important to consider that dopaminergic neurons from substantia nigra have highly ramified striatal axonal arborizations and exhibit high plasticity. Thus, the surviving dopaminergic neurons appear to be able to supply striatal innervation by compensatory axonal sprouting, resulting in a relative preservation of axon terminals in the striatum despite a larger loss of nigral dopaminergic neurons (Paß et al., 2024). Furthermore, the analysis of TH-positive neurons is more direct (number of soma) than the analysis of TH terminals (integrated density of staining), which may limit the precision of terminal loss assessment. Considering this, and with the PD model being validated, we showed that 1 mg/kg of AD-16 given orally daily for 7 days after the model onset conferred neuroprotection, by reducing TH loss in both the SNc and the striatum, in addition of alleviating motor impairment. Our findings highlighted AD-16 as a promising treatment for PD, and taken together with the previous studies, indicate the compound’s capability to relieve the pathology of neuroinflammatory-associated disorders.

One of the main players involved in the inflammatory response of neurodegenerative diseases is the microglia (Kwon and Koh, 2020). This cell presents a very diverse and complex morphology that can vary from a highly ramified and branched morphology, usually associated with a surveillant state, to a round, ameboid, and more phagocytic state. Pro-inflammatory signals released from degenerating neurons may trigger the activation of microglia cells to the latter (Stefanova, 2022; Lull and Block, 2010), although it is not clear whether this activation takes place only after neuronal death starts or if it could be one of the primary causes that sets off cell degeneration. We recently reported morphological alterations in microglia from 6-OHDA-injected mice such as a decrease in the number of endpoints and in the branch length, besides less complexity, increase in cell density, and reduction in circularity, among other parameters that indicate an ameboid-like and pro-inflammatory morphology (Ferreira et al., 2024b). It is important to note that microglia have a very heterogeneous morphology that varies according to sex, age, region, and state of health (Vidal-Itriago et al., 2022; Tan et al., 2020). Nonetheless, active microglia cause a deleterious cycle in PD and other neurodegenerative disorders by producing pro-inflammatory cytokines and other factors, such as IL-1α, IL-1β, IL-6, IL-10, TNF-α, reactive oxygen species (ROS) and nitric oxide (NO), all of which can provoke apoptosis and cell death (Nagatsu and Sawada, 2005). Increase in pro-inflammatory cytokines have been reported before in the 6-OHDA model, such as TNF-α, IFN-γ, IL-1β, IL-2, IL-6, TGF-β1, IL-4 (Nagatsu et al., 2000; Haas et al., 2016; Antunes et al., 2020).

Others have reported that AD-16 was able to reduce IL-1β expression induced by LPS *in vivo* (Sun et al., 2020), restore IL-4 and IL-6 levels in a mouse model of Alzheimer’s disease (Zhou et al., 2016), and reduce IL-1β and TNF-α levels in the brain of ischemic mice (Zhang et al., 2024). In addition, the treatment also reduced the microglia cell area that was larger in the Alzheimer’s group, indicating a change in the morphology (Sun et al., 2020). We found that AD-16 treatment reduced microglia density, restored its morphology and reduced the pro-inflammatory cytokine levels (IL-1α, IL-1β, IL-6, and TNF-α) in the 6-OHDA PD model. We notice that the differences were more pronounced in the striatal region, as the number of endpoints and some cytokines (IL-1β and IL-6) were not improved by AD-16 in substantia nigra. It is important to consider the methodological challenges involved when studying such a small nucleus as the substantia nigra, as it is hard to delimit the boundaries from the near-about areas. This may explain some of the conflicting results observed. In addition, we cannot exclude the hypothesis that these cytokines are being resealed by other cell types, such as astrocytes, monocytes, and macrophages (Dinarello, 2018; Guan et al., 2021; Aliyu et al., 2022). Finally, we need to consider the fact that higher doses or a different treatment time window might be necessary to induce benefits in this brain region. As another limitation of this study, we cannot observe alpha-synuclein inclusions in the 6-OHDA model. Microglia is known to play a role in alpha-synuclein processing and degradation. The excess of alpha-synuclein observed in PD brains can cause microglial activation, oxidative stress, and contribute to the pro-inflammatory state (Deyell et al., 2023). The effect of AD-16 on some alpha-synucleinopathy model is certainly essential as a next step.

Even though the exact mechanism of action through which AD-16 can reduce inflammation is not clear, there are several hypotheses that could explain its capacity. AD-16 has a similar chemical structure to a p38α MAPK (mitogen-activated protein kinase) inhibitor. The p38a MAPK pathway is known to be activated both in patients and animal models of Alzheimer’s disease and is correlated to an increase of pro-inflammatory cytokines levels, while the use of inhibitors of this cascade blocks this production (Munoz et al., 2007). An inhibitor, chemically similar to AD-16, successfully suppressed the increase of IL-1β and TNF-α levels in an Alzheimer’s disease model (Munoz et al., 2007), suggesting that AD-16 could also modulate this pathway. Besides that, AD-16 was shown to alter lysosomal position and enhance LAMP1 expression in microglial cells, suggesting an improvement in lysosomal function and, thus, enhancing microglia clearance capacity of dysfunctional proteins and debris that contribute to neuroinflammation (Sun et al., 2020).

## 5 Conclusion

We showed here that AD-16 treatment, a neuroinflammation modulator, alleviated motor impairment, reduced neurodegenera -tion, and microglia activation in the 6-hydroxydopamine (6-OHDA) mouse model of PD. These novel findings, associated with the previous reports on the safety and feasibility of the compound, indicate AD-16 as a new promising potential therapeutic approach for PD.

## Data Availability

The raw data supporting the conclusions of this article will be made available by the authors, without undue reservation.
